# Supplementary material for: An exclusive human milk diet for very low birth weight newborns—A cost-effectiveness and EVPI study for Germany
Source: PLoS One. 2019 Dec 30;14(12):e0226496. doi: 10.1371/journal.pone.0226496 (PMC6936873; doi:10.1371/journal.pone.0226496)
Supplement: S1 File — This file contains a detailed list of all model parameters. (PDF) [file pone.0226496.s001.pdf]

Appendix for “The cost-effectiveness of an exclusive human milk diet for very low birth weight new-borns in Germany”

Part 1: Medical input parameters of the model.

Name	Mean	SD	Low	High	Probabilistic value	Source	Study period	Country	Comment
**Cohort size**	9519		8136	10903			2012-2016	GER	Weighted mean
<500gr	475		396	554		[1–5]	2012-2016	GER	yearly cases
500-749gr	1540		1227	1854		[1–5]	2012-2016	GER	yearly cases
750-999gr	2004		1741	2267		[1–5]	2012-2016	GER	yearly cases
1000-1249gr	2166		1868	2464		[1–5]	2012-2016	GER	yearly cases
1250-1499gr	3335		2905	3765		[1–5]	2012-2016	GER	yearly cases
**- Probability NEC**	5.70%	0.18%	4.94%	6.54%	Dirichlet(gamma(1;542.8))		1998-2001	USA	Weighted mean
<500gr	7.19%	0.47%	6.29%	8.18%	Dirichlet(gamma(1;34.15))	[6]	1998-2001	USA	adjusted by RR for P(NEC|Sepsis)
500-749gr	8.67%	0.51%	7.69%	9.74%	Dirichlet(gamma(1;133.54))	[6]	1998-2001	USA	
750-999gr	6.86%	0.43%	6.03%	7.77%	Dirichlet(gamma(1;137.49))	[6]	1998-2001	USA	
1000-1249gr	5.48%	0.41%	4.71%	6.34%	Dirichlet(gamma(1;118.75))	[6]	1998-2001	USA	
1250-1499gr	3.56%	0.31%	2.98%	4.23%	Dirichlet(gamma(1;118.86))	[6]	1998-2001	USA	
**-- Probability NEC surgical**	29.03%	0.87%	21.81%	28.72%	beta(787.71;1925.29)		2012-2016	GER	Weighted mean
<500gr	52.71%	3.82%	44.93%	60.38%	beta(89.47;80.29)	[9]	2012-2016	GER	Redistributed across birth weights according to Jeschke 2016
500-749gr	43.79%	1.92%	39.98%	47.65%	beta(291.93;374.78)	[9]	2012-2016	GER	
750-999gr	30.35%	1.75%	26.93%	33.94%	beta(208.32;478.15)	[9]	2012-2016	GER	
1000-1249gr	21.01%	1.67%	17.80%	24.51%	beta(124.56;468.19)	[9]	2012-2016	GER	
1250-1499gr	12.15%	1.34%	9.64%	15.06%	beta(72.11;521.19)	[9]	2012-2016	GER	
**--- Death background**	Same as mortality of VLBW with No Complication								
**--- Death due to NEC surgical**	14.65%	1.07%	6.08%	15.68%	beta(159.85;931.15)		2000-2011	GER	Weighted mean
<500gr	19.53%	4.27%	6.71%	32.35%	beta(16.6;68.4)	[10]	2000-2011	GER	
500-749gr	18.39%	2.03%	13.01%	23.78%	beta(67.14;297.86)	[10]	2000-2011	GER	
750-999gr	14.94%	1.97%	10.78%	19.10%	beta(48.85;278.15)	[10]	2000-2011	GER	
1000-1249gr	7.34%	1.96%	3.50%	11.19%	beta(12.93;163.07)	[10]	2000-2011	GER	
1250-1499gr	6.03%	2.05%	1.64%	10.42%	beta(8.08;125.92)	[10]	2000-2011	GER	
**---- Probability BPD|NEC surgical**	7.24%	0.27%	6.73%	7.78%	beta(689.33;8829.07)	[1–5]	2012-2016	GER	Average yearly cases between 2012 and 2016; Only cases with gestational age of >24 weeks
<500gr	52.08%	7.21%	37.19%	66.71%	beta(24.48;22.52)	[12]	2013-2014	KOR	Moderate and severe BPD

500-749gr	48.06%	2.84%	42.38%	53.78%	beta(148.52;160.48)	[12]	2013-2014	KOR	
750-999gr	40.84%	2.15%	36.60%	45.19%	beta(213.59;309.41)	[12]	2013-2014	KOR	
1000-1249gr	30.24%	1.96%	26.42%	34.27%	beta(165.7;382.3)	[12]	2013-2014	KOR	
1250-1499gr	18.25%	1.63%	15.13%	21.70%	beta(101.82;456.18)	[12]	2013-2014	KOR	
**---- Probability SBS|NEC surgical**	19.39%	1.85%	15.87%	23.31%	beta(88.81;369.19)	[13]	2002-2005	USA	
<500gr	18.60%	2.15%	15.87%	23.31%	beta(60.81;266.19)	calc	2002-2005	USA	Cole 2008 found no significant difference in risk for SBS and birth weight when controlling for NEC.
500-749gr	18.60%	2.15%	15.87%	23.31%	beta(60.81;266.19)	calc	2002-2005	USA	
750-999gr	18.60%	2.15%	15.87%	23.31%	beta(60.81;266.19)	calc	2002-2005	USA	
1000-1249gr	21.37%	3.58%	15.87%	23.31%	beta(27.79;102.21)	calc	2002-2005	USA	
1250-1499gr	21.37%	3.58%	15.87%	23.31%	beta(27.79;102.21)	calc	2002-2005	USA	
**---- Probability RoP|NEC surgical**	8.97%	0.25%	7.42%	10.83%	beta(1178.93;11967.07)		2008-2012	GER	Weighted mean
<500gr	26.74%	1.71%	20.61%	33.97%	beta(179.66;492.34)	[11]	2008-2012	GER	adjusted by RR for P(RoP|NEC)
500-749gr	31.71%	1.00%	27.90%	35.84%	beta(680.42;1465.58)	[11]	2008-2012	GER	
750-999gr	9.55%	0.56%	7.68%	11.72%	beta(263.8;2499.2)	[11]	2008-2012	GER	
1000-1249gr	1.76%	0.24%	1.05%	2.78%	beta(53.96;3006.04)	[11]	2008-2012	GER	
1250-1499gr	0.27%	0.08%	0.07%	0.68%	beta(11.99;4489.01)	[11]	2008-2012	GER	
**---- Probability CP|NEC surgical**	23.84%	3.25%	17.68%	30.92%	beta(40.76;130.24)	[7, 8]	>1974 & 2002-2004	USA	No weight stratification; Sonntag 2007 ignored due to low case numbers
**---- Probability Neuro|NEC surgical**	0.39285714	0.09229619	0.21504283	0.59423179	beta(10.61;16.39)	[7]	2002-2004	USA	No weight stratification
**---- Probability No complication|NEC surgical**	Complementary probability other complications/morbidities assuming independene, i.e. 1-P(BPD)*P(RoP)*P(SBS)*P(CP)*P(NI).								
**-- Probability NEC medical**	Complementary probability to the Probability of NEC surgical								
**--- Death background**	Same as mortality of VLBW with No Complication								
**--- Death due to NEC medical**	Same as mortality for NEC surgical								
**---- Probability BPD|NEC medical**	Same as BPD probability for NEC surgical								
**---- Probability RoP|NEC medical**	Same as ROP probability for NEC surgical								
**---- Probability CP|NEC medical**	11.64%	2.33%	7.44%	17.09%	beta(21.88;166.12)	[7, 8]	>1974 & 2002-2004	USA	No weight stratification; Sonntag 2007 ignored due to low case numbers
**---- Probability Neuro|NEC medical**	23.08%	6.75%	11.13%	39.33%	beta(8.77;29.23)	[7]	2002-2004	USA	No weight stratification
**---- Probability No complication|NEC medical**	Complementary probability other complications/morbidities assuming independene, i.e. 1-P(BPD)*P(RoP)*P(CP)*P(NI).								

**- Probability Sepsis**	12.67%	0.12%	12.14%	13.21%	Dirichlet(gamma(1;1206.05))	calc	2012-2016	GER	Weighted mean
<500gr	36.23%	1.18%	33.91%	38.59%	Dirichlet(gamma(1;172))	[9]	2012-2016	GER	adjusted by RR for P(NEC|Sepsis)
500-749gr	19.74%	0.33%	19.11%	20.39%	Dirichlet(gamma(1;304.09))	[9]	2012-2016	GER	
750-999gr	20.06%	0.33%	19.42%	20.71%	Dirichlet(gamma(1;401.96))	[9]	2012-2016	GER	
1000-1249gr	5.91%	0.15%	5.61%	6.22%	Dirichlet(gamma(1;128.02))	[9]	2012-2016	GER	
1250-1499gr	6.00%	0.16%	5.70%	6.31%	Dirichlet(gamma(1;199.98))	[9]	2012-2016	GER	
**Death background**	General Mortality of VLBW without major morbidity								
**Death Sepsis**	17.46%	2.76%	12.33%	23.64%	beta(32.83;155.17)	[14]	2010	GER	
**---- Probability BPD|Sepsis**	Same BPD probability as for NEC surgical								
**---- Probability RoP|Sepsis**	2.80%	0.14%	2.32%	3.38%	beta(368.02;12777.98)		2008-2012	GER	Weighted mean
<500gr	8.35%	1.07%	6.43%	10.60%	beta(56.09;615.91)	[11]	2008-2012	GER	adjusted by RR for P(RoP|!NEC)
500-749gr	9.90%	0.64%	8.71%	11.19%	beta(212.41;1933.59)	[11]	2008-2012	GER	
750-999gr	2.98%	0.32%	2.40%	3.66%	beta(82.35;2680.65)	[11]	2008-2012	GER	
1000-1249gr	0.55%	0.13%	0.33%	0.87%	beta(16.85;3043.15)	[11]	2008-2012	GER	
1250-1499gr	0.08%	0.04%	0.02%	0.21%	beta(3.74;4497.26)	[11]	2008-2012	GER	
**---- Probability CP|Sepsis**	19.53%	2.44%	6.71%	32.35%	beta(51.56;212.44)	[7, 8]	>1974 & 2002-2004	USA	No weight stratification; Sonntag 2007 ignored due to low case numbers
**---- Probability Neuro|Sepsis**	35.92%	3.07%	29.91%	42.27%	beta(87.64;156.36)	[7]	2002-2004	USA	No weight stratification
**---- Probability No complication|Sepsis**	Complementary probability other complications/morbidities assuming independene, i.e. 1-P(BPD)*P(RoP)*P(CP)*P(NI).								
**- Probabiltiy NEC + Sepsis**	2.28%	0.12%	1.83%	2.82%	Dirichlet(gamma(1;217.16))				Weighted mean
<500gr	7.12%	0.63%	5.93%	8.47%	Dirichlet(gamma(1;33.83))				adjusted by RR for P(NEC|Sepsis)
500-749gr	4.68%	0.39%	3.95%	5.50%	Dirichlet(gamma(1;72.09))				
750-999gr	3.70%	0.32%	3.09%	4.40%	Dirichlet(gamma(1;74.22))				
1000-1249gr	0.85%	0.17%	0.56%	1.25%	Dirichlet(gamma(1;18.5))				
1250-1499gr	0.56%	0.13%	0.34%	0.86%	Dirichlet(gamma(1;18.52))				
**NEC surgical + Sepsis**	Same as proportion for NEC surgical without sepsis								
**Death background**	General Mortality of VLBW without major morbidity								
**Death due to NEC**	Mortality of NEC surgical combined with mortality of Sepsis								
**BPD**	Probability of BPD|NEC combined with probability BPD|Sepsis								
**SBS**	Same as probability for SBS for NEC without sepsis								
**RoP**	Probability of RoP|NEC combined with probability RoP|Sepsis								

**CP**	21.43%	10.97%	4.66%	50.80%	beta(2.79;10.21)	[7]	2002-2004	USA	
**Neuro**	50.00%	13.36%	23.04%	76.96%	beta(6.5;6.5)	[7]	2002-2004	USA	
**No complication**	Complementary probability other complications/morbidities assuming independence.								
**NEC medical + Sepsis**	Same as proportion for NEC medical without sepsis								
**Death background**	General Mortality of VLBW without major morbidity								
**Death due to NEC**	Mortality of NEC without NEC combined with mortality of Sepsis								
**BPD**	BPD of NEC combined with BPD of Sepsis								
**RoP**	RoP of NEC combined with RoP of Sepsis								
**CP**	20.00%	8.94%	5.73%	43.66%	beta(3.8;15.2)	[7]	2002-2004	USA	
**Neuro**	45.00%	11.12%	23.06%	68.47%	beta(8.55;10.45)	[7]	2002-2004	USA	
**No complication**	Complementary probability other complications/morbidities assuming independene, i.e. 1-P(BPD)*P(RoP)*P(CP)*P(NI).								
**No NEC or sepsis**	79.35%		81.08%	77.43%	Dirichlet(gamma(1;7553.39))				complementary probability other complications/morbidities; assuming independene
<500gr	49.46%		53.86%	44.77%	Dirichlet(gamma(1;234.82))				
500-749gr	66.91%		69.25%	64.38%	Dirichlet(gamma(1;1030.48))				
750-999gr	69.38%		71.46%	67.13%	Dirichlet(gamma(1;1390.32))				
1000-1249gr	87.75%		89.12%	86.19%	Dirichlet(gamma(1;1900.33))				
1250-1499gr	89.88%		90.99%	88.60%	Dirichlet(gamma(1;2997.44))				
**Death background**	12.73%	0.41%	11.65%	13.91%	beta(838.92;5752.05)				
<500gr	56.70%	4.91%	51.90%	61.60%	beta(57.08;43.59)	[11]	2008-2012	GER	180 day survival without major morbidity
500-749gr	37.80%	1.92%	35.70%	40.00%	beta(241.9;398.06)	[11]	2008-2012	GER	180 day survival without major morbidity
750-999gr	16.40%	1.09%	15.10%	17.90%	beta(190.45;970.86)	[11]	2008-2012	GER	180 day survival without major morbidity
1000-1249gr	6.20%	0.57%	5.40%	7.20%	beta(110.45;1671.05)	[11]	2008-2012	GER	180 day survival without major morbidity
1250-1499gr	3.10%	0.32%	2.60%	3.60%	beta(90.01;2813.51)	[11]	2008-2012	GER	180 day survival without major morbidity
**BPD**	Same BPD probability as for NEC surgical								
**RoP**	2.80%	0.14%	2.32%	3.38%	beta(368.02;12777.98)		2008-2012	GER	Weighted mean
<500gr	8.35%	1.07%	6.43%	10.60%	beta(56.09;615.91)	[11]	2008-2012	GER	adjusted by RR for P(RoP|!NEC)
500-749gr	9.90%	0.64%	8.71%	11.19%	beta(212.41;1933.59)	[11]	2008-2012	GER	
750-999gr	2.98%	0.32%	2.40%	3.66%	beta(82.35;2680.65)	[11]	2008-2012	GER	
1000-1249gr	0.55%	0.13%	0.33%	0.87%	beta(16.85;3043.15)	[11]	2008-2012	GER	
1250-1499gr	0.08%	0.04%	0.02%	0.21%	beta(3.74;4497.26)	[11]	2008-2012	GER	
**CP**	14.96%	1.25%	12.57%	17.60%	beta(120.85;687.15)	[7]	2002-2004	USA	
**Neuro**	22.00%	1.46%	19.19%	25.02%	beta(177.78;630.22)	[7]	2002-2004	USA	

**No complication**	Complementary probability other complications/morbidities assuming independene, i.e. 1-P(BPD)*P(RoP)*P(CP)*P(NI).								
**RR (NEC total)**	0.400	0.141	0.528	0.357	lognormal(-0.992;0.14)	[15, 16]	2008/2010	USA	
**RR (NEC surgical)**	0.452	0.468	1.131	0.152	lognormal(-0.79;0.47)	[15, 16]	2008/2010	USA	
**RR (Sepsis)**	0.680	0.080	0.795	0.664	lognormal(-0.39;0.08)	[15, 16]	2008/2010	USA	
**RR (Sepsis + NEC)**	0.272	0.011	0.420	0.274	lognormal(-1.30;0.01)				Combined
**RR (mortality)**	0.716	0.117	0.902	0.790	lognormal(-0.33;0.12)	[15. 16]	2008/2010	USA	
**RR (BPD)**	0.849	0.048	0.933	0.846	lognormal(-0.16;0.05)	[16]		USA	
**RR (RoP)**	0.551	0.184	0.789	0.515	lognormal(-0.60;0.18)	[16, 17]		USA	
**RR (CP)**	0.90	-	1.00	1.00	1.00				1 assumed in scenario analyses
**RR (Neurological)**	0.90	-	1.00	1.00	1.00				

Part 2a: Economic input parameter values.

Name	Mean	SD	Low	High	Probabilistic value	Source	Study period	Country	Comment
**Costs EHDM**	6,812 €		4,415 €	7,569 €	Not applicable				
<500gr	13,015 €		8,622 €	14,221 €	Not applicable				
500-749gr	11,895 €		7,863 €	13,221 €	Not applicable				
750-999gr	9,106 €		5,973 €	10,348 €	Not applicable	Prolacta® Biosciences			
1000-1249gr	5,208 €		3,359 €	5,760 €	Not applicable				
1250-1499gr	3,246 €		2,129 €	3,317 €	Not applicable				
**Donor milk (litres)**	3.71		2.93	3.28	Not applicable	[18]			
<500gr	7.08		5.67	7.18	Not applicable	[18]			
500-749gr	6.49		5.19	5.95	Not applicable	[18]			
750-999gr	4.97		3.97	4.11	Not applicable	[18]			Only used in scenario analysis.
1000-1249gr	2.81		2.25	1.65	Not applicable	[18]			
1250-1499gr	1.77		1.41	1.95	Not applicable	[18]			
**Price donor milk (per litre)**	65.00 €		60.00 €	90.00 €	Not applicable	Personal communication (Milk bank Leipzig)	2018	GER	
**Costs BOV**	223 €		223	223	Not applicable				
<500gr	612 €		612	612	Not applicable				
500-749gr	444 €		444	444	Not applicable				
750-999gr	276 €		276	276	Not applicable				Only used in scenario analysis.
1000-1249gr	108 €		108	108	Not applicable				
1250-1499gr	108 €		108	108	Not applicable				
**LOS Factor**									

<500gr	1.49	0.04	1.43	1.58	gamma(1151;0.0013)	[9]	2002-2016	GER	
500-749gr<br>*750-999gr*	1.34	0.01	1.32	1.35	gamma(14960.29;0.0001)	[9]	2002-2016	GER	Available for categories <500gr, 500-999gr and 1000-1499gr
1000-1249gr<br>*1250-1499gr*	0.75	0.01	0.74	0.77	gamma(7187.27;0.0001)	[9]	2002-2016	GER	
**LOS NEC**	31.25	46.96	31.20	31.30	gamma(0.44;70.57)	[19]	2006-2016	GER	
**LOS NEC mortality**	20.81	47.00	20.22	21.40	gamma(0.2;106.18)	[19]	2006-2016	GER	
**LOS Sepsis**	8.39	6.89	8.38	8.39	gamma(1.48;5.66)	[19]	2006-2016	GER	
**LOS Sepsis mortality**	5.26	13.30	5.10	5.42	gamma(0.16;33.68)	[19]	2006-2016	GER	
**LOS NEC + Sepsis**	31.25	46.96	31.20	31.30	gamma(0.44;70.57)	[19]	2006-2016	GER	
**LOS NEC + Sepsis mortality**	13.03					[19]			Weighted average of NEC and sepsis.
**LOS no complication**									
<500gr<br>*500-749gr*<br>*750-999gr*	74.15	61.29	74.15	74.15	gamma(1.46;50.67)	[19]	2006-2016	GER	
1000-1249gr<br>*1250-1499gr*	20.49	21.56	20.49	20.49	gamma(0.90;22.68)	[19]	2006-2016	GER	
**LOS no complication mortality**	11.67	32.48	11.66	11.68	gamma(0.13;90.37)		2006-2016	GER	
**LOS RoP**	12.64	24.01	12.61	12.67	gamma(0.28;45.60)	[19]	2006-2016	GER	
**LOS SBS**	29.13	34.84	29.00	29.26	gamma(0.70;41.67)	[19]	2006-2016	GER	
**LOS BPD**	25.57	34.19	25.53	25.60	gamma(0.56;45.73)	[19]	2006-2016	GER	
**Basecase**	3,376 €	18 €	3,342 €	3,461 €	gamma(36916.37;0.09)	[20]	2017	GER	
**Direct Costs Cerebral Palsy**	232,703 €	79,504 €	76,874 €	471,616 €	gamma(8.57;27163.25)	[21]	2017	GER	
**Direct Costs Neurological**	94,220 €	8,316 €	77,920 €	112,403 €	gamma(128.37;733.99)	[21]	2017	GER	
**Life-time earnings**	396,543 €	39,654 €	318,820.25 €	474,264.95 €	gamma(100;3965.43)	[22]	2017	GER	
**Reduced productivity CP**	48.1%		63.9%	42.6%	beta(12.98;13.48)	[23]	-	GER	
**Reduced productivity Neurological**	60.2%		77.8%	27.9%	beta(9.95;6.18)	[23]	-	GER	
**Friction period**	92	14.29	64	98	gamma(41.47;2.22)	[24]	2015	GER	

Part 2b: Diagnosis related groups (DRGs) used for cost calculations

Health state	Birth weight	DRG	Cost weight	Mean LOS	1st day	1st day	Deduction	Surcharge

					deduction	surcharge		
No Complication	<500gr	P61B	36.02	104.40	34	122	1.02	0.34
	500-749gr	P61D	30.07	88.90	29	107	1.00	0.34
	750-999gr	P62B, P62C	23.28	71.42	22	89	0.97	0.32
	1000-1249gr	P63Z	9.30	42.00	13	60	0.66	0.21
	1250-1499gr	P64Z	6.91	32.70	10	51	0.62	0.21
NEC or Sepsis	<500gr	P61A	47.51	102.70	33	121	1.35	0.45
	500-749gr	P61C	46.40	114.50	37	132	1.18	0.43
	750-999gr	P62A	30.58	80.70	26	99	1.10	0.37
	1000-1249gr	P03C	12.03	47.40	15	65	0.00	0.00
	1250-1499gr	P03C	12.03	47.40	15	65	0.00	0.00
NEC + Sepsis	<500gr	P61A	47.51	102.70	33	121	1.35	0.45
	500-749gr	P61C	46.40	114.50	37	132	1.18	0.43
	750-999gr	P62A	30.58	80.70	26	99	1.10	0.37
	1000-1249gr	P03A, P03B	16.49	54.91	16	72	0.90	0.29
	1250-1499gr	P03A, P03B	16.49	54.91	16	72	0.90	0.29
Mortality	<500gr	P61E	6.19	11.50	0	24	0.00	0.53
	500-749gr	P61E	6.19	11.50	0	24	0.00	0.53
	750-999gr	P62D	9.72	17.00	5	1000	1.58	0.00
	1000-1249gr	P60A	0.72	1.40	0	1000	0.00	0.00
	1250-1499gr	P60A	0.72	1.40	0	1000	0.00	0.00
SBS	overall	G04Z, G50Z, G67A, G67B	0.89	5.84	1.45	12.21	0.33	0.08
RoP	overall	C63Z, C14Z	0.58	3.75	1	8.25	0.27	0.08
BPD	overall	E69D	0.48	3.00	1	6.00	0.24	0.11

References

1. IQTIG. Bundesauswertung zum Erfassungsjahr 2016 - Neonatologie: Qualitätsindikatoren; 12.07.2017.

2. IQTIG. Bundesauswertung zum Erfassungsjahr 2015 - Neonatologie: Qualitätsindikatoren; 07.07.2016.

3. AQUA-Institut GmbH. Qualitätsreport 2014; 2015.

4. AQUA-Institut GmbH. Qualitätsreport 2013; 2014.

5. AQUA-Institut GmbH. Qualitätsreport 2012; 2013.

6. Guillet R, Stoll BJ, Cotten CM, Gantz M, McDonald S, Poole WK, Phelps DL. Association of H2-blocker therapy and higher incidence of necrotizing enterocolitis in very low birth weight infants. Pediatrics. 2006;117:e137-42. doi:10.1542/peds.2005-1543.

7. Martin CR, Dammann O, Allred EN, Patel S, O'Shea TM, Kuban KCK, Leviton A. Neurodevelopment of extremely preterm infants who had necrotizing enterocolitis with or without late bacteremia. J Pediatr. 2010;157:751-6.e1. doi:10.1016/j.jpeds.2010.05.042.

8. Rees CM, Pierro A, Eaton S. Neurodevelopmental outcomes of neonates with medically and surgically treated necrotizing enterocolitis. Arch Dis Child Fetal Neonatal Ed. 2007;92:F193-8. doi:10.1136/adc.2006.099929.

9. NRZ. KISS Krankenhaus-Infektions-Surveillance-System: Modul NEO-KISS Referenzdaten; 10.05.2017.

10. Schwab F, Zibell R, Piening B, Geffers C, Gastmeier P. Mortality due to bloodstream infections and necrotizing enterocolitis in very low birth weight infants. Pediatr Infect Dis J. 2015;34:235–40. doi:10.1097/INF.0000000000000532.

11. Jeschke E, Biermann A, Günster C, Böhler T, Heller G, Hummler HD, Bührer C. Mortality and Major Morbidity of Very-Low-Birth-Weight Infants in Germany 2008-2012: A Report Based on Administrative Data. Front Pediatr. 2016;4:23. doi:10.3389/fped.2016.00023.

12. Jo HS, Cho KH, Cho S-I, Song ES, Kim BI. Recent Changes in the Incidence of Bronchopulmonary Dysplasia among Very-Low-Birth-Weight Infants in Korea. J Korean Med Sci. 2015;30 Suppl 1:S81-7. doi:10.3346/jkms.2015.30.S1.S81.

13. Cole CR, Hansen NI, Higgins RD, Ziegler TR, Stoll BJ. Very low birth weight preterm infants with surgical short bowel syndrome: incidence, morbidity and mortality, and growth outcomes at 18 to 22 months. Pediatrics. 2008;122:e573-82. doi:10.1542/peds.2007-3449.

14. Stichtenoth G, Demmert M, Bohnhorst B, Stein A, Ehlers S, Heitmann F, et al. Major contributors to hospital mortality in very-low-birth-weight infants: data of the birth year 2010 cohort of the German Neonatal Network. Klin Padiatr. 2012;224:276–81. doi:10.1055/s-0032-1306344.

15. Abrams SA, Schanler RJ, Lee ML, Rechtman DJ. Greater mortality and morbidity in extremely preterm infants fed a diet containing cow milk protein products. Breastfeed Med. 2014;9:281–5. doi:10.1089/bfm.2014.0024.

16. Hair AB, Peluso AM, Hawthorne KM, Perez J, Smith DP, Khan JY, et al. Beyond Necrotizing Enterocolitis Prevention: Improving Outcomes with an Exclusive Human Milk-Based Diet. Breastfeed Med. 2016;11:70–4. doi:10.1089/bfm.2015.0134.

17. O'Connor DL, Kiss A, Tomlinson C, Bando N, Bayliss A, Campbell DM, et al. Nutrient enrichment of human milk with human and bovine milk-based fortifiers for infants born weighing <1250 g: a randomized clinical trial. Am J Clin Nutr. 2018;108:108–16. doi:10.1093/ajcn/nqy067.

18. Texas Children's Hospital. Feeding Protocol with Cream. 23rd ed.; 2017.

19. Statistisches Bundesamt. Krankenhausstatistik. 2017. http://www.gbe-bund.de/oowa921-install/servlet/oowa/aw92/dboowasys921.xwdevkit/xwd_init?gbe.isgbetol/xs_start_neu/&p_aid=3&p_aid=88658613&nummer=702&p_sprache=D&p_indsp=-&p_aid=99250666#SOURCES.

20. GKV Spitzenverband. Bundesbasisfallwert (BBFW). 2018. https://www.gkv-spitzenverband.de/krankenversicherung/krankenhaeuser/budgetverhandlungen/bundesbasisfallwert/bundesbasisfallwert.jsp.

21. Scholz S, Meszaros K, Fassbender RM, Welte R, Greiner W, Koerber F. The Cost Of Illness For Invasive Meningococcal B Disease In Germany. Value in Health. 2017;20:A785. doi:10.1016/j.jval.2017.08.2290.

22. Statistisches Bundesamt. Wirtschaftsrechnungen 2015: Laufende Wirtschaftsrechnungen: Einkommen, Einnahmen und Ausgaben privater Haushalte. Wiesbaden; 17.01.2017.

23. Deutscher Bundestag. Verordnung zur Durchführung des § 1 Abs. 1 und 3, des § 30 Abs. 1 und des § 35 Abs. 1 des Bundesversorgungsgesetzes: Versorgungsmedizin-Verordnung - VersMedV; 10. Dezember 2008 (BGBl. I S. 2412).

24. Bundesagentur für Arbeit. Arbeitsmarkt 2015: Arbeitsmarktanalyse für Deutschland, West- und Ostdeutschland. Nürnberg; August 2016.
