# Supplementary material for: An exclusive human milk diet for very low birth weight newborns—A cost-effectiveness and EVPI study for Germany
Source: PLoS One. 2019 Dec 30;14(12):e0226496. doi: 10.1371/journal.pone.0226496 (PMC6936873; doi:10.1371/journal.pone.0226496)
Supplement: Attachment — Submitted filename: Rebuttal_letter.docx [file pone.0226496.s002.docx]

**Editor Comments:**

**1. Thank you for your funding statement; "Both authors, SMS and WG, wish to disclose that the research on which our manuscript is based was funded by an unrestricted research grant by Prolacta BioSciences (https://www.prolacta.com/). The funder had no influence on the study design, data collection and analysis, decision to publish and the preparation of the manuscript. "**

**We note that you received funding from a commercial source: Prolacta BioScience**

**Please provide an amended Competing Interests Statement that explicitly states this commercial funder, along with any other relevant declarations relating to employment, consultancy, patents, products in development, marketed products, etc.**

**Within this Competing Interests Statement, please confirm that this does not alter your adherence to all PLOS ONE policies on sharing data and materials by including the following statement: "This does not alter our adherence to PLOS ONE policies on sharing data and materials.” (as detailed online in our guide for authors http://journals.plos.org/plosone/s/competing-interests). If there are restrictions on sharing of data and/or materials, please state these. Please note that we cannot proceed with consideration of your article until this information has been declared.**

**Please include your amended Competing Interests Statement within your cover letter. We will change the online submission form on your behalf.**

**Please know it is PLOS ONE policy for corresponding authors to declare, on behalf of all authors, all potential competing interests for the purposes of transparency. PLOS defines a competing interest as anything that interferes with, or could reasonably be perceived as interfering with, the full and objective presentation, peer review, editorial decision-making, or publication of research or non-research articles submitted to one of the journals. Competing interests can be financial or non-financial, professional, or personal. Competing interests can arise in relationship to an organization or another person. Please follow this link to our website for more details on competing interests: http://journals.plos.org/plosone/s/competing-interests**

*Thank you for giving such detailed advice on how to proceed on this issue. We amended the cover letter by the following competing interest statement:*

*“The presence of the funding from a commercial source does not alter our adherence to PLOS ONE policies on sharing data and materials. The authors declare that there are no other competing interests with regard to the research conducted for this manuscript.”*

*Please let us know if there is any other information needed to proceed.*

**2. Major English language corrections are required, there are too many problems which need fixing before it can be considered suitable for further review. I suggest hiring a language expert who should essentially rewrite the whole manuscript so that it becomes easy to read so that average reader can understand the meaning.**

*Thank you for your comment. A native speaker from the US has proof-read and corrected the paper to improve the readability of the paper.*

**3. Manuscript is statistics heavy, tables are too long, results presented in a very complex manner which are hard to understand for average reader of PLOS one, kindly shorten data, every single detail need not be provided but salient features are shown in tables, cutting back on some figures and tables will help.**

*Thank you for this comment. We would like to keep the figures and the tables in the manuscript in their current state to make our research reproducible and to adhere to the standard reporting in health economic evaluation.*

**4. It should be very clear in results and discussion section that this study results are valid only to the local German population and cannot be applied universally since the NEC rate as well as cost of human milk fortifier are highly variable between centers. Likewise hospital charges are much different then actual cost of the product making this work difficult to use.**

*Thank you for this comment. We expanded the discussion to address the issue of limited transferability of the results to other health care systems and countries. The discussion section now starts as follows:*

*“The results of this modelling study need to be interpreted in the light of several limitations. First of all, the study population of the clinical studies on EHMD efficacy is from the US and does not entirely match the German model population by the clinical complications and birth weight. The results for the sub-groups of the model population corresponding to the birth weights of the clinical studies were very similar to the overall cost-effectiveness (€35,464 vs. €34,016 per LYG, respectively), but it remains unclear if the effectiveness estimates can be transferred to new-borns with birth weights below 750g or above 1,250g. The transferability of our results is also limited with regard to other countries due to the specific NEC rates, the German reimbursement scheme for inpatient costs and the cost of human milk and fortifiers in Germany.“*

**2. Review Comments to the Author**

**Reviewer #1:**

*Thank you very much for taking the time to review our paper and thank you for your constructive feedback. We are grateful you even specified the lines where changes need to be made. Please see below our responses on your comments, which where all included in the new version of the manuscript.*

**Line 34 : Please provide the expansion of LYG and ICER here**

*Thank you very much, we added the expansion of LYG and ICER.*

**Line 48 : need to be BPD instead of BDP**

*Thank you for noticing this. The typo has been corrected.*

**Line 108-109 : what is meant by the statement"in cost scenario for EHMD a ratio of 30/70 is assumed using Prolact+8 (with a ratio of 40/60)". Why is it assumed as 30/70 when its 40/60**

*Sorry for this relict of a previous version. The correct ratio is 40/60 and the sentence has been changed accordingly. It now reads as follows:*

*“In the lower scenario, Prolact+4 is used with a ratio of 20/80ml and in the high cost scenario for EHMD a ratio of 40/60 is assumed using Prolact+8.”*

**Line 115-116 : If there are no available data for the effectiveness of EHMD on neurological sequelae, based on what criteria was the RR of 0.90 assumed**

*There is no direct evidence for the effectiveness of EHMD on neurological sequelae as the manifestation of a neurological impairment will only become evident when the children are at around school age. But there are studies suggesting that NEC and sepsis are associated with a higher probability of neurological impairment and reducing NEC and sepsis thus also leads to a reduction in neurological impairments (please see references below). We therefore included – from our perspective – a conservative value of 0.90 for neurological sequelae. Please let us know if this acceptable in your opinion.*

Roze et al. 2011 Functional Impairments at School Age of Children With Necrotizing Enterocolitis or Spontaneous Intestinal Perforation, Pediatric Research 70(6):619-625.

van der Ree et al. 2011, Functional impairments at school age of preterm born children with late-onset sepsis, Early Human Development 87:821-826.

Ehrenkranz et al. 2006, Growth in the Neonatal Intensive Care Unit Influences Neurodevelopmental and Growth Outcomes of Extremely Low Birth Weight Infants, Pediatrics 117(4):1253-1261.

**Line 136-138 : What was the process used to calculate the LOS data based on birth weight? What is the adjustment factor and how was it arrived at?**

*Unfortunately, there is no data source available that would allow estimating the LOS for the different conditions (NEC, sepsis, etc.) stratified by age. However, there are two different data sources available that give information about the marginal distributions of the LOS by birth weight and the LOS for each of the conditions, respectively. We used the data source giving the LOS by birth weight to calculate the ratio of the LOS for each birth weight category, compared to the LOS for all new-borns with a birth weight between 500g and 1499g.The LOS for the different conditions were then multiplied by this factor. We added some more information on the calculation and the sentence now reads as follows:*

*“As the ICD-specific LOS data were not available stratified by birth weight, adjustment factors have been calculated from another German data source on LOS of very low birth weight infants by calculating the ratio of the LOS of the different birth weight categories and the overall LOS of all new-borns with a birth weight between 500g and 1499g.”*

**Line 160 : Put WTP in parenthesis here so that the reader can recognize what it stands for later in the manuscript.**

*The abbreviation WTP for willingness-to-pay has been added.*

**Line 177 - its showing both Table 2Table 1. which one is it? Table 2?**

*Sorry, this seems to be a problem with the automated numbering in Word. We removed all automated fields. It should read as Table 2.*

**Table 2 : Please add "for the base case" in the legend.**

*We amended the table caption as you suggested.*

**Reviewer #2:**

**1. Table or Figure should be self-explained. The abbreviation should be noted, e.g. what are DSA, PSA and so on.**

*Thank you for this suggestion. We added the explanations of the abbreviations used in the table and figures in the respective captions.*

**2. Authors mentioned that the health state specific probabilities of complication were either directly calculated or estimated from other studies. It would be good to indicate which is from direct calculation and which is estimated from other studies in the presentation of results/tables.**

*Thank you for this comment. We included a sentence in the methods section that states the probabilities which were estimated from other studies. The sentence now reads: “When this was not possible (i.e. for the probabilities of NEC alone and RoP given NEC and sepsis, respectively), relative risks have been calculated from studies [26, 27] and were applied to German datasets to retrieve health state specific complication probabilities.” We also added asterisks to parameters in table 1 for which other studies were used.*

**3. Some estimates, e.g. health state specific probabilities, the efficacy of EHMD and so on, were not directly calculated based on the sample but estimated from other studies. For those from other studies, please comment on how compatible or generalizable to the German population.**

*You are correct, that the transferability of the results from the other studies is limited. We amended the limitations section in the discussion to put more emphasis on this point. The discussion starts as follows:*

*“The results of this modelling study need to be interpreted in the light of several limitations. First of all, the study population of the clinical studies on EHMD efficacy is from the US and does not entirely match the German model population by the clinical complications and birth weight. The results for the sub-groups of the model population corresponding to the birth weights of the clinical studies were very similar to the overall cost-effectiveness (€35,464 vs. €34,016 per LYG, respectively), but it remains unclear if the effectiveness estimates can be transferred to new-borns with birth weights below 750g or above 1,250g. The transferability of our results is also limited with regard to other countries due to the specific NEC rates, the German reimbursement scheme for inpatient costs and the cost of human milk and fortifiers in Germany.“*
